# Overcoming Central Venous Occlusion: The First HeRO Graft Procedure in the Baltic States

**DOI:** 10.3390/jcm15218347

**Published:** 2026-10-27

**Authors:** Monika Vitkauskaitė, Dalia Bogomolovienė, Andrius Drobnys, Simonas Jonas Norvydas, Laurynas Rimševičius, Diana Sukackienė, Marius Miglinas

**Affiliations:** 1 Clinic of Gastroenterology, Nephro-Urology and Surgery, Faculty of Medicine, Institute of Clinical Medicine, Vilnius University, 03101 Vilnius, Lithuania; 2 Center for Vascular Reconstructive and Endovascular Surgery, Vilnius University Hospital Santaros Klinikos, 08406 Vilnius, Lithuania; 3 Faculty of Medicine, Vilnius University, 03101 Vilnius, Lithuaniadiana.sukackiene@santa.lt (D.S.);

**Keywords:** HeRO graft, central venous occlusion, end-stage kidney disease, hemodialysis, arteriovenous graft, case report

## Abstract

**Background**: Central venous occlusive disease is a major challenge in hemodialysis patients who have exhausted conventional vascular access options and may lead to long-term dependence on tunneled central venous catheters. The Hemodialysis Reliable Outflow (HeRO) graft provides an alternative by bypassing obstructed central veins and enabling catheter-free upper-extremity vascular access. **Case presentation**: We report the first HeRO graft implantation in Lithuania in a 78-year-old woman with end-stage kidney disease and extensive central venous occlusive disease. Previous arteriovenous fistulas had failed because of thrombosis and severe venous hypertension, while prolonged catheter dependence was complicated by recurrent infections, dislodgements, and multiple catheter exchanges. The HeRO venous outflow component was implanted through the existing tunneled catheter access and positioned in the right atrium. It was connected to an early-cannulation expanded polytetrafluoroethylene graft anastomosed to the brachial artery. Completion fistulography demonstrated unobstructed flow through the system into the right atrium. The procedure was technically successful, and the graft was successfully cannulated for hemodialysis 24 h after implantation, avoiding the need for temporary catheter placement. **Conclusions**: The HeRO graft may provide a valuable catheter-sparing option for carefully selected hemodialysis patients with severe central venous occlusive disease and no feasible conventional upper-extremity access. This case demonstrates its technical feasibility in a complex vascular access setting and highlights the potential role of an early-cannulation graft when immediate catheter removal is necessary.

## 1. Introduction

Reliable vascular access is essential for the survival of patients with end-stage kidney disease (ESKD) undergoing maintenance haemodialysis [1]. Native arteriovenous fistulas (AVFs) are the preferred vascular access because they provide the best long-term patency and are associated with the lowest rates of infection and thrombosis, followed by arteriovenous grafts (AVGs) when AVF creation is not feasible [2]. However, establishing and preserving functional access remains a major clinical challenge, particularly as advances in healthcare have prolonged patient survival [1]. Adequate venous outflow is fundamental to the creation and long-term function of arteriovenous (AV) access; as suitable outflow options become progressively more central, vascular access creation becomes increasingly complex and may ultimately become impossible because of central venous stenosis or occlusion. Central venous stenosis or occlusion may impair venous return, resulting in access dysfunction, venous hypertension, and severe upper-extremity oedema [3,4,5,6]. As vascular access options become exhausted, many patients become dependent on long-term tunneled dialysis catheters, which are associated with increased risks of thrombosis, catheter-related bloodstream infections, repeated interventions, hospitalization, and substantial healthcare costs [7,8].

For patients who have exhausted conventional vascular access options because of severe central venous outflow obstruction, the Hemodialysis Reliable Outflow (HeRO) graft has emerged as an alternative to long-term tunneled dialysis catheters. Approved by the U.S. Food and Drug Administration (FDA) in 2008 and subsequently receiving CE Mark approval in Europe in 2013, the HeRO graft is a fully subcutaneous vascular access system that is specifically referenced in the 2019 Kidney Disease Outcomes Quality Initiative (KDOQI) vascular access guidelines for appropriately selected patients [1,9,10]. Although classified as a graft, it differs from conventional AVG by lacking a venous anastomosis. Instead, the system consists of an arterial polytetrafluorethylene graft anastomosed to the brachial artery and a venous outflow component, which is inserted percutaneously through the internal jugular or subclavian vein with its tip positioned in the right atrium. The arterial graft and venous outflow component are connected subcutaneously using a titanium connector, creating continuous blood flow directly from the artery to the right atrium while bypassing obstructed central veins. As the entire system is fully subcutaneous, the HeRO graft provides definitive catheter-free vascular access while reducing the infection risk associated with external catheter components [3,11,12].

We report the first HeRO graft implantation in the Baltic States in a patient with end-stage kidney disease who had exhausted conventional vascular access options and whose only remaining alternative for maintenance hemodialysis was long-term central venous catheter dependence.

## 2. Case Report

### 2.1. Case Presentation and Vascular Access History

A 78-year-old woman with end-stage kidney disease secondary to chronic tubulointerstitial nephritis attributed to analgesic nephropathy underwent HeRO graft implantation in June 2026. Her medical history was significant for hypertension and colorectal cancer, treated with sigmoid colon resection and adjuvant chemotherapy, with complete remission at the time of presentation.

Urgent maintenance hemodialysis was initiated in October 2021 via a central venous catheter (CVC). A native arteriovenous fistula (AVF) was created in the left upper extremity in November 2021 but thrombosed after four months. Subsequently, a proximal right upper-arm AVF was constructed in March 2022. However, the AVF could not be cannulated because of severe ipsilateral upper-extremity soft tissue oedema resulting from impaired venous outflow, and it was surgically ligated in June 2022. Haemodialysis was therefore continued via a tunneled CVC (tCVC). Angiographic and cross-sectional imaging demonstrated extensive bilateral central venous occlusive disease, including occlusion of the right and left brachiocephalic and subclavian veins.

Conventional percutaneous endovascular recanalisation of the central venous occlusions had previously been attempted twice at another hospital, but both attempts were unsuccessful. Given these previous failures, another conventional endovascular recanalisation attempt was not considered a favourable option.

During the following years, the patient remained dependent on CVCs but experienced recurrent catheter-related infections and catheter dislodgements, necessitating multiple catheter exchanges, including femoral venous access.

Lower-extremity vascular access was also evaluated. Although pedal pulses were palpable bilaterally, duplex ultrasonography demonstrated circumferential calcification of both superficial femoral arteries with a high calcium burden. Given these findings, creation of lower-extremity haemodialysis access was considered to carry an unacceptably high risk of lower-limb ischaemic complications, including dialysis access-related steal syndrome, and was therefore not pursued.

Due to the previous upper-extremity access failures, unsuccessful attempts at conventional central venous recanalisation, recurrent catheter-related complications, extensive bilateral central venous occlusion, and limited suitability for lower-extremity access, a HeRO graft was selected as the preferred option.

### 2.2. Preoperative Planning and Two-Stage Access Strategy

Computed tomography venography was performed before the intervention to define the extent of the central venous disease and to plan the access strategy (Supplementary Video S1). The study confirmed extensive bilateral central venous occlusive disease involving both brachiocephalic and subclavian veins and was used to assess the feasibility and trajectory of the planned intervention. Because of the extent and complexity of the central venous occlusions, the intervention was planned as a two-stage procedure.

The first stage consisted of sharp recanalisation to establish a reliable central venous route for subsequent HeRO graft implantation. Femoral venous access was obtained, and a Bernstein catheter was advanced centrally and positioned within the superior vena cava as an intravascular fluoroscopic target. Using the catheter as a landmark, sharp percutaneous recanalisation into the superior vena cava was performed under fluoroscopic guidance. After successful entry into the superior vena cava, a guidewire was advanced into the right atrium, confirming a traversable route. A tunneled central venous catheter was then placed through this newly established access. This first-stage procedure served both therapeutic and procedural-planning purposes: it provided functional haemodialysis access and, importantly, established a stable route to the right atrium that could subsequently be used for definitive HeRO graft implantation.

The second stage consisted of definitive HeRO graft implantation using the previously established tCVC tract.

### 2.3. HeRO Graft Implantation

**Figure 1:**
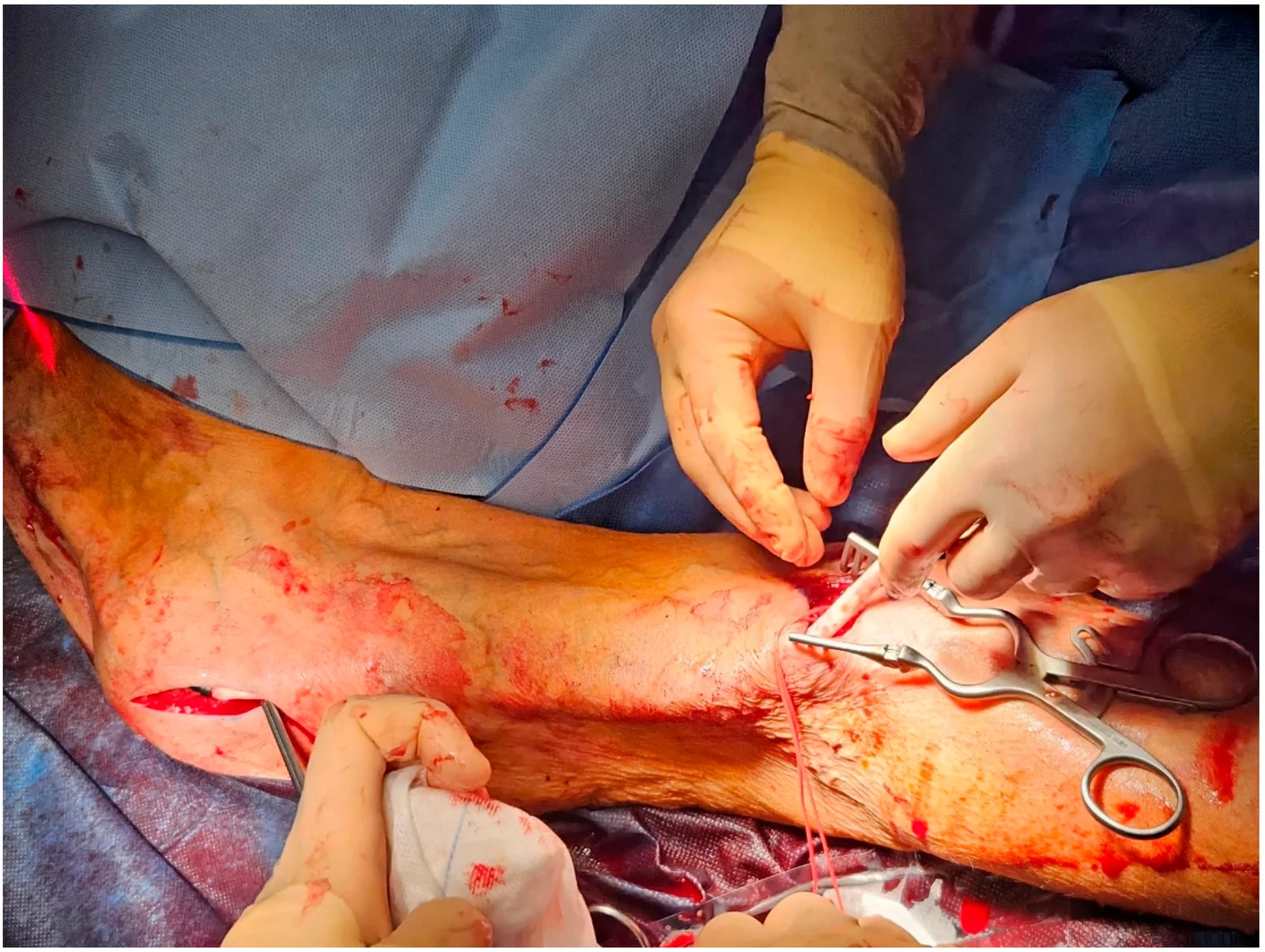
Intraoperative view during HeRO graft implantation in the right upper extremity.

The procedure was performed under general anaesthesia, and 2 g of intravenous cefazolin was administered for antibiotic prophylaxis. The brachial artery was exposed at the cubital fossa (Figure 1).

The previously implanted tCVC was exposed and divided. A 0.035-inch hydrophilic guidewire was advanced through the catheter lumen into the inferior vena cava. The hydrophilic guidewire was then exchanged for an Amplatz Super Stiff guidewire to provide adequate support for subsequent device advancement.

After removal of the tCVC, the established tract was predilated using an 8 × 60 mm angioplasty balloon.

### 2.4. Balloon-Assisted Advancement of the Venous Outflow Component

Despite predilatation of the tract, advancement of the HeRO venous outflow component (VOC) proved technically difficult. To provide additional internal support, a 5 mm angioplasty balloon was introduced into the lumen of the VOC. The balloon was inflated within the VOC, providing temporary internal support and increasing the pushability of the component during advancement over the stiff guidewire.

The balloon-supported VOC was advanced under fluoroscopic guidance towards the right atrium. Once the desired position had been achieved, the balloon was deflated and removed.

This balloon-assisted manoeuvre represented an off-label technical modification used to facilitate controlled advancement of the VOC through the resistant pre-existing catheter tract.

### 2.5. Arterial Graft Implantation and Completion Imaging

The VOC was connected to a 4–7 mm tapered Flixene early-cannulation ePTFE graft using a Super HeRO Adapter. The graft was tunnelled subcutaneously and anastomosed end-to-side to the brachial artery using a continuous 6-0 polypropylene (Prolene) suture. An early-cannulation ePTFE graft was selected to permit prompt haemodialysis after removal of the existing tCVC and to avoid the need for temporary catheter placement.

The final position of the VOC was confirmed intraoperatively by fluoroscopy. Completion fistulography demonstrated brisk flow through the HeRO circuit with unobstructed outflow into the right atrium.

**Figure 2:**
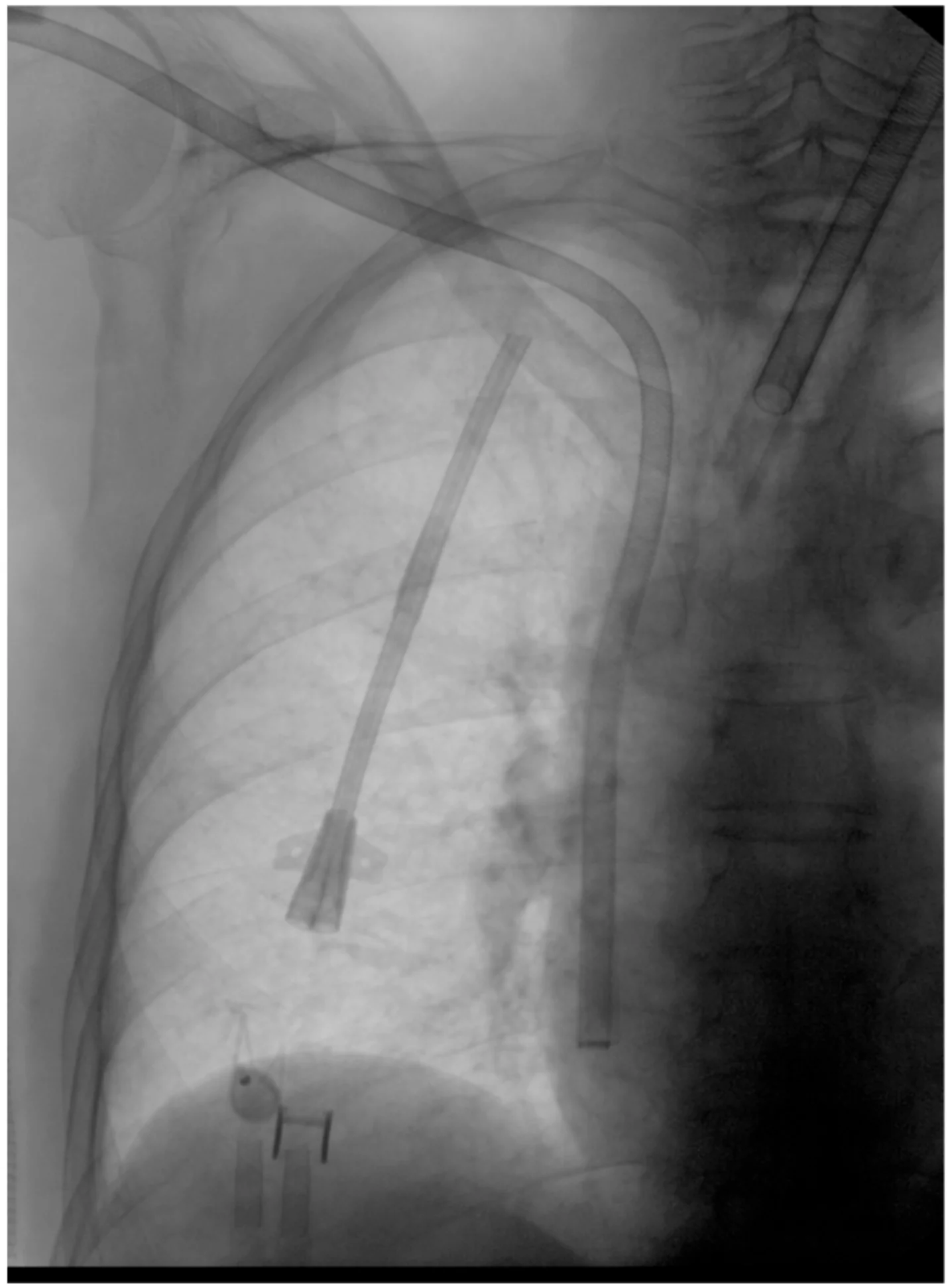
Intraoperative fluoroscopic image demonstrating the final position of the HeRO Venous Outflow Component in the right atrium.

No quantitative intraoperative graft-flow measurement was performed. Postoperative chest radiography was not considered necessary because satisfactory positioning of the VOC had already been confirmed intraoperatively by fluoroscopy (Figure 2) and completion fistulography.

**Figure 3:**
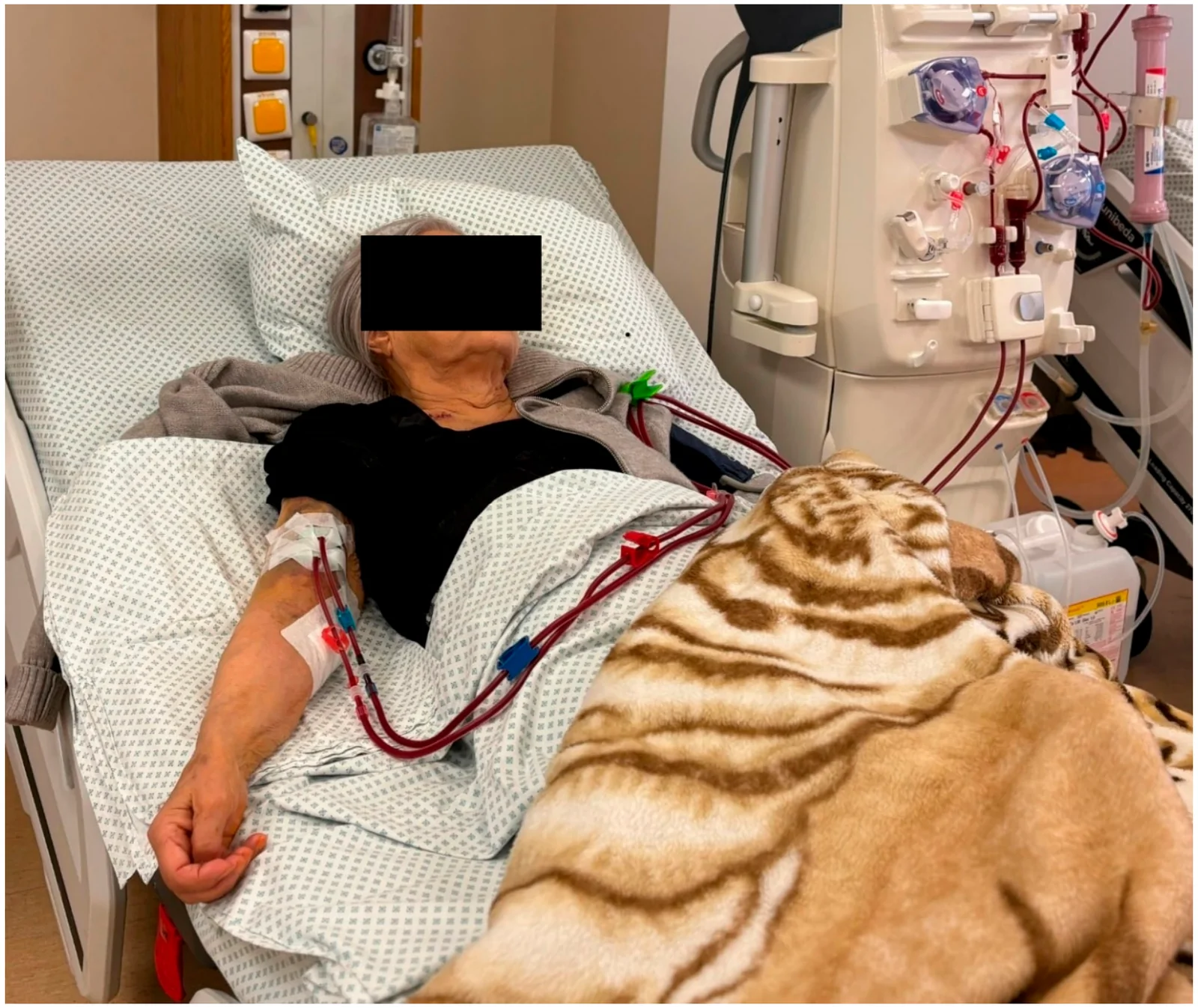
Successful haemodialysis using the HeRO graft following implantation.

Twenty-four hours after surgery, the graft was successfully cannulated for the first planned haemodialysis session (Figure 3), with a blood flow rate (Qb) of 300 mL/min. No technical difficulties were encountered during cannulation or dialysis.

### 2.6. Short-Term Follow-Up

At 3 months after implantation, the HeRO graft remained patent and functional. An ultrasound evaluation of the VOC demonstrated preserved blood flow without mural filling defects; the arterial anastomosis was patent, with no evidence of stenosis. Dialysis adequacy remained satisfactory, with a Kt/V of 1.35, compared with 1.10 before HeRO graft implantation.

## 3. Discussion

This case highlights the potential role of the HeRO graft in patients with complex vascular access failure, in whom central venous outflow obstruction precludes conventional upper-extremity AV access. In our patient, HeRO graft implantation provided an alternative to long-term tunneled catheter dependence after conventional access options had been exhausted. The case also represents the first reported experience with HeRO graft implantation in the Baltic States.

The HeRO graft occupies a distinct position within the vascular access algorithm and is primarily intended for use in patients with moderate to severe central venous occlusion and/or stenosis [13]. In such patients, conventional AVF or AVG creation may be unsuccessful or may result in severe venous hypertension [14], while continued dependence on a tCVC exposes the patient to cumulative risks of infection, biofilm formation, thrombosis, and loss of additional venous access sites [15]. By bypassing the obstructed central venous segment and providing direct outflow into the central circulation, the HeRO graft offers a means of maintaining upper-extremity AV access without an external catheter component [10].

Available evidence suggests that the principal clinical advantage of the HeRO graft over tCVCs is a reduction in catheter-associated morbidity [10,16]. Previous cohort studies have reported substantially lower bloodstream infection rates with HeRO grafts compared with tCVCs, together with improved dialysis adequacy and reduced catheter dependence [1,16]. Moreover, Katzman et al. found that all HeRO-related bacteremias occurred during the bridging period when a tCVC was still implanted before HeRO graft incorporation [16]. These previously reported advantages provided part of the rationale for avoiding continued catheter dependence in our patient, who had experienced recurrent catheter-related infections and multiple catheter exchanges. However, given the single-patient design and three-month follow-up, the present case cannot establish reductions in infection risk, catheter-related morbidity, or other comparative clinical benefits of the HeRO graft.

Nonetheless, HeRO graft implantation should not be considered equivalent to creation of a conventional native AVF but rather as an alternative access option [10,17]. Published series demonstrate that maintenance of HeRO graft patency frequently requires secondary interventions, with a reported intervention rate of 1.5 per patient-year and thrombosis accounting for up to 82% of interventions, frequently requiring thrombectomy [1,18]. Despite declining primary patency from 60% at 6 months to 48.8% at 12 months and 42.9% at 24 months, secondary patency remained 86.7% at 24 months, highlighting the importance of repeated interventions in maintaining long-term access function [18]. More recent evidence continues to demonstrate the substantial reintervention burden associated with HeRO grafts while supporting their role in maintaining long-term vascular access in patients with otherwise limited access options [19]. Although infection rates are lower than those associated with tCVCs, graft-related infection remains a potential complication of HeRO access [1]. Steal syndrome is an important complication, with reported rates of 1.4–2.6% in larger series [1,16], although Wallace et al. observed a substantially higher incidence of 22%, particularly among women [20]. Rare device-related complications, including migration of the outflow component into the right ventricle and graft separation during percutaneous thrombectomy, have also been reported [21,22]. These potential complications and the frequent need for secondary interventions must be balanced against the benefits of reduced catheter dependence, underscoring the importance of careful patient selection and continued surveillance following HeRO graft implantation.

The extent of central venous disease in our patient is particularly noteworthy. Cross-sectional imaging demonstrated extensive bilateral central venous occlusion involving the right brachiocephalic vein and left jugular-subclavian confluence. Furthermore, a previous upper-extremity AVF had resulted in severe venous hypertension and arm oedema, necessitating ligation. These findings made another conventional upper-extremity AV access unsuitable and illustrate the clinical scenario for which the HeRO system was developed. Successful implantation allowed restoration of catheter-independent upper-extremity haemodialysis access despite severely compromised conventional venous outflow.

An additional clinically relevant aspect of this case was the ability to establish functional access rapidly after implantation. In contrast to earlier HeRO series, in which a bridging tCVC was commonly required during graft incorporation [16], the use of an early-cannulation graft allowed haemodialysis to resume within 24 h without placement of an additional temporary catheter. This was particularly relevant in our patient because the existing tCVC had to be removed during HeRO implantation, while further catheter exposure was undesirable given the history of recurrent catheter-related infections and severely limited central venous access. Thus, although the HeRO graft itself is an established vascular access option, this case highlights the practical value of combining a complex two-stage access strategy with an early-cannulation graft to minimize continued catheter dependence in a high-risk patient.

Although the immediate procedural and functional outcome was favourable, the short duration of follow-up is an important limitation of this report. Long-term surveillance will be required to assess primary and secondary patency, the need for access-related interventions, infectious complications, and sustained dialysis adequacy. Consequently, this case cannot establish the long-term effectiveness of the HeRO graft but demonstrates its technical feasibility and immediate clinical utility in a patient with otherwise exhausted conventional vascular access options.

## 4. Conclusions

In conclusion, the HeRO graft may provide a valuable catheter-sparing option for carefully selected haemodialysis patients with severe central venous occlusive disease and no feasible conventional upper-extremity AV access. The present case demonstrates the technical feasibility and short-term functionality of this strategy in a patient with otherwise exhausted conventional vascular access options and severely compromised central venous outflow.

## Data Availability

The data supporting the article are available from Monika Vitkauskaitė on reasonable request.

## Supplementary Materials

The following supporting information can be downloaded at: https://www.mdpi.com/article/10.3390/jcm15218347/s1, Video S1: Preoperative CT venography demonstrating extensive bilateral central venous occlusive disease. On the right, the internal jugular-subclavian confluence and the right brachiocephalic vein were not visualized, consistent with occlusion/obliteration. More proximal portions of the right subclavian, axillary, and internal jugular veins remained contrast-opacified. Extensive collateral venous pathways were present in the right upper thoracic region, supporting chronic central venous obstruction. On the left, the brachiocephalic vein demonstrated only fragmentary contrast opacification, while a distinct internal jugular-subclavian confluence could not be identified. The left axillary and internal jugular veins remained contrast-opacified proximally.

## Abbreviations

The following abbreviations are used in this manuscript:

AV	Arteriovenous
AVF	Arteriovenous fistula
AVG	Arteriovenous graft
CE	Conformité Européenne
CVC	Central venous catheter
ePTFE	Expanded polytetrafluoroethylene
ESKD	End-stage kidney disease
FDA	United States Food and Drug Administration
HeRO	Haemodialysis Reliable Outflow
KDOQI	Kidney Disease Outcomes Quality Initiative
tCVC	Tunneled central venous catheter
VOC	Venous Outflow Component
